# Metastatic Adenocarcinoma of the Lung Presenting as a Soft Tissue Mass

**DOI:** 10.1155/2021/8856503

**Published:** 2021-01-06

**Authors:** B. Yglesias, M. Brooker, R. DeVito, A. Swiger

**Affiliations:** ^1^Department of Surgery, Western Reserve Health Education, Warren, Ohio, USA; ^2^American University of Antigua, Antigua and Barbuda; ^3^Northeast Ohio Medical University, Rootstown, Ohio, USA

## Abstract

Lung cancer is the leading cause of cancer death in the United States, with more than 230,000 new cases, and approximately 150,000 deaths estimated for 2018. Lung cancer most commonly metastasizes to the brain, liver, lungs, bone, and adrenal system; however, there have been several cases of spread to soft tissues, with an incidence rate of approximately 0.75-9%. The objective of this case report is to highlight an unusual presentation of metastatic adenocarcinoma of the lung. In this case report, patient presented with a 3 × 3 cm soft tissue mass on the back. The mass was slowly growing but had become more painful and wished to have it excised. Preoperatively, the mass was suspected to be a sebaceous cyst but intraoperatively had deep attachments and other suspicious findings. Pathology had a positive immunoprofile for metastatic adenocarcinoma favoring a lung primary. Given this presentation of metastases, the prognosis is poor with a survival time decreasing to around 5 months. Overall, this case reinforces the importance of sending all soft tissue masses for final pathology with accurate labeling and the importance of immunohistochemical testing in aiding the identification of the primary.

## 1. Introduction

Lung cancer is the leading cause of cancer death in the United States, with more than 230,000 new cases, and approximately 150,000 deaths estimated for 2018 [1]. With a survival rate of only 18%, it is important to recognized unusual presentations of metastases early. Adenocarcinoma typically constitutes 40% of these cases [2]. The histology of invasive lung adenocarcinoma consists primarily of 5 different histological patterns: leptic, acinar, papillary, micropapillary, and solid with mucin [3]. If metastases are present, the survival time after diagnosis drops to 5 months [4].

Lung cancer preferentially metastasizes to the brain, liver, lungs, bone, and adrenal system [4]. Although these are the most common sites of metastases, there have been several cases of spread to soft tissues, with an incidence rate of approximately 0.75-9% [5]. The most common location is the skeletal muscle of the thigh [6]. Often if there is a soft tissue aspect, it presents with pain and weakness of the affected area [7]. An unusual presentation of painless, itchy, red swollen, or even ruptured nodule has also been reported.

Diagnostic imaging of these masses is widely nonspecific as they most commonly resemble soft tissue sarcomas clinically, thus making biopsy necessary for definitive diagnosis [8]. While these soft tissue masses often present in the late stages of the disease, they may occasionally develop either prior to or concurrently with the initial diagnosis of malignancy [9]. In this case study, we present a soft tissue mass that has a positive immunoprofile for metastatic focus of adenocarcinoma with micropapillary features favoring a lung primary.

## 2. Case Report

This patient is an 81-year-old male with a past medical history of COPD, CKD, and atrial fibrillation and a history of pneumonia treated by his primary care physician and pulmonary medicine specialist 3 months prior to presentation. Patient also had a 46-pack year smoking history who quit in 1998. He presented to the office with a mass of questionable soft tissue origin in his back that was causing discomfort and pain. The lesion was reported by the patient as a firm, slow-growing, and bothersome for him to lean back in his chair or car seat. Lying on his back was also painful. Initial examination the mass was 3 × 3 cm and mildly tender to palpation with no accompanying lymphadenopathy, redness, or discharge. After discussion of the risks, benefits, and alternative options, the shared physician-patient relationship decided to undergo surgical excision of the back mass. Informed consent was obtained.

The patient underwent LMAC anesthesia in the prone position with local application of 1% Xylocaine with epinephrine. An elliptical incision of the skin overlying the mass measuring was performed for grossly negative surgical margins. Circumferentially, the mass was taken down to the fascia and musculature. There were suspicious attachments of the mass to the surrounding musculature and fascia requiring excision for an en bloc resection. This was performed using cautery. The mass was removed, oriented, labelled, and sent to pathology.

The tissue sample which measured 3.7 × 2.8 × 2.5 cm was stained at the margins with black ink and was noted to have a somewhat circumscribed fleshy appearing area measuring 2.3 × 2 × 1.5 cm. Microscopically, this appeared to extend to the stained ink margins. The sample was sectioned in blocks A1-A9 and subsequently immunostained. Block A5 displayed positive staining for TTF-1, CK7, and CDX-2, with negative CK20. This immunostaining leads to a diagnosis of metastatic adenocarcinoma with micropapillary features from a lung primary. The sample was noted to be an incomplete excision with transection of the mass at the peripheral margin.

The patient was notified of the results, and oncology was consulted. Oncology recommends a staging workup and reexcision of the mass. The patient declined any surgical reexcision. The patient also refused to undergo further staging workup or medical or surgical treatment.

## 3. Discussion

As with all types of lung cancer, the patient's long smoking history put him at strong risk of developing the lung primary. However, preoperatively, the mass on the back was thought to be a benign sebaceous cyst. The patient denied any fever, chills, shortness of breath, hemoptysis, weight loss, or any concerning signs or symptoms for lung cancer. If the patient's history were concerning for lung cancer, a different preoperative approach would have been considered. This would include initially a chest radiograph. If concerning features are present, then proceed with a CT of the chest/abdomen/pelvis to assess the primary lesion and for adenopathy and metastasis. Blood work would also be obtained including CBC, BMP, and LFTs for liver metastasis, calcium, and alkaline phosphatase. If the patient reports headaches, a brain MRI can be considered. If the patient has elevated alkaline phosphatase or reports bone pain, a bone scan can be obtained for bone metastasis. Unfortunately, in this case report, the patient denied any symptoms concerning lung cancer, so no preoperative imaging or workup was obtained.

This case presents the atypical manifestation of pulmonary adenocarcinoma metastasis as a soft tissue mass. Soft tissue metastasis is highly unusual in cancers of any origin with a prevalence of 1.8%. Of those resulting from a lung primary, adenocarcinoma is the histological type most commonly reported [10]. TTF-1 immunostain is found almost exclusively in pulmonary adenocarcinoma [11]. In a 2002 study, the combination of CK7 positive and CK20 negative was evident in 95% of the cases of metastatic adenocarcinomas of the lung being followed [12]. All of these diagnostic immunostains characteristics were present in this case.

There is evidence that pulmonary adenocarcinoma with micropapillary features, such as seen in this patient, may benefit from adjuvant chemotherapy after complete excision [13]. Despite this possibility, often when these metastases are present, the neoplasm has already advanced and metastasized to other organ systems. Even with cancer therapy, the prognosis of lung cancer with skin metastases is very poor, with an average survival from the time of skin metastasis diagnosis being only around 5 months [4]. In our case report, chemotherapy and reexcision were not pursued due to patient wishes.

Accurate radiographic and pathologic staging with both prognostic and therapeutic implications should be performed. In the past, MRI has been supported as a tool for diagnostic and treatment planning, despite its nonspecific nature. More recent studies have shown that F-18 FDG PET/CT scanning is more sensitive for both skin and soft tissue metastases [14]. If our patient underwent further treatment, a PET/CT scan would have been recommended to localize other areas of metastasis. For isolated metastatic lesions, surgical excision alone may be sufficient or paired with radiotherapy, if the primary malignancy has been treated [5]. As our pathology sample contained positive gross margins, further surgical excision would be required if this was the desired approach of treatment. However, the patient declined any further investigation or management.

## 4. Conclusion

This is a case report of a soft-tissue mass of unknown origin causing pain and discomfort and noted on physical examination. Surgical intervention was chosen due to the location and superficial nature of the lesion. Upon excision, histological findings of the mass were indicative of metastatic pulmonary adenocarcinoma with micropapillary features with positive margins. With this presentation of metastatic pulmonary adenocarcinoma, the prognosis was poor and the patient declined to undergo further management. The case report highlights the importance of sending all soft tissue masses to pathology regardless of the preoperative diagnosis.
